# The Neuronal Encoding of Oral Fat by the Coefficient of Sliding Friction in the Cerebral Cortex and Amygdala

**DOI:** 10.1093/cercor/bhy213

**Published:** 2018-08-31

**Authors:** Edmund T Rolls, Tom Mills, Abigail B Norton, Aris Lazidis, Ian T Norton

**Affiliations:** 1 Oxford Centre for Computational Neuroscience, Oxford, England; and University of Warwick, Department of Computer Science, Coventry, England; 2 School of Chemical Engineering, University of Birmingham, Birmingham, England

**Keywords:** fat texture, food texture, insula, nutrition, orbitofrontal cortex

## Abstract

Fat in the diet contributes to the pleasant mouthfeel of many foods, but overconsumption may contribute to obesity. Here we analyze what properties of fat in the mouth are sensed, by analyzing the responses of neurons in the macaque insular taste cortex, and two areas to which it projects the orbitofrontal cortex where the pleasantness of fat is represented, and the amygdala. We discovered that the firing rate responses of these fat-responsive neurons are correlated with the coefficient of sliding friction (CSF) and not with viscosity which reflects food thickness. Other, not fat-sensitive, neurons encoded viscosity and not the CSF. Neuronal population analyses confirmed that fat-responsive neurons conveyed information about the CSF but not about viscosity. Conversely the viscosity-sensitive neuronal population conveyed information about viscosity but not about the CSF. This new understanding of the representation of oral fat in the cerebral cortex and amygdala opens the way for the systematic development of foods with the pleasant mouthfeel of fat, together with ideal nutritional content and has great potential to contribute to healthy eating and a healthy body weight.

## Introduction

Fat in the diet is an important source of energy, provides fatty acids that are essential (Vannice and Rasmussen 2014), and can contribute to the pleasantness of food in the mouth (Rolls 2011, 2016c). A mechanism has evolved that enables fat in food to be detected based on oral texture. This has been shown in pioneering studies in which single neurons in the macaque primary taste cortex in the anterior insula (Verhagen et al. 2004), in the secondary taste cortex in the orbitofrontal cortex (Rolls et al. 1999; Verhagen et al. 2003), and in the amygdala (Kadohisa et al. 2005b), have been shown to respond selectively to fat texture in the mouth (Kadohisa et al. 2005a). These neurons respond to the texture of fat in that they respond to food oils such as coconut oil, safflower oil, and vegetable oil, to emulsions such as dairy cream, and also to mineral oil (a pure hydrocarbon), and to silicone oil. These neurons do not respond to fatty acids such as linoleic acid (LiA) or lauric acid (LaA), and their responses are not correlated with taste properties such as sweet, salt, bitter, sour, and umami, nor with oral temperature (Rolls et al. 1999; Kadohisa et al. 2005a). Consistent results have been found in humans with functional magnetic resonance neuroimaging (de Araujo and Rolls 2004; Grabenhorst et al. 2010). This evidence thus shows that these populations of neurons in primates encode fat and fatty emulsions such as cream by their texture (Rolls 2011, 2016c). However, the property of texture that is being encoded by fat-sensitive neurons is not known, though it is clear that it is not viscosity (Verhagen et al. 2003; Kadohisa et al. 2005a), and indeed separate neurons encode oral viscosity (Rolls et al. 2003). The hypothesis investigated here is whether the neurons categorized as having responses to fat and nonfat oils in previous neurophysiological investigations (Verhagen et al. 2003, 2004; Kadohisa et al. 2005a, 2005b) have responses that are correlated with the coefficient of sliding friction (CSF) rather than with viscosity.

We therefore performed the research described here in which the physical properties of the stimuli used in the original neurophysiological experiments (Rolls et al. 2003; Verhagen et al. 2003, 2004; Kadohisa et al. 2005b) were analyzed, to determine whether a physical property could be identified with which the neuronal responses were correlated. The physical parameters investigated were the CSF and the viscosity. The CSF is the force required to slide two surfaces divided by the force normal to the surfaces (Ludema 1996). This is also known as kinetic or dynamic friction.

## Methods

### Stimuli

The stimuli that were used in the neurophysiological investigations (Verhagen et al. 2003, 2004; Kadohisa et al. 2005a, 2005b) are shown in Table 1 and included a viscosity series of the food thickening agent carboxymethylcellulose with nominal viscosities measured with a Brookfield rheometer in the original experiments of 10, 100, 1000 and 10 000 centiPoise (1 Pa.s = 1000 cP); safflower, coconut and vegetable oil, and single cream (18% fat); silicone oil (either 10, 100, and 1000 cP or 280 cP); mineral oil; and distilled water. The carboxymethylcellulose series was included in the investigations to assess whether any neurons had responses to the thickness of the food, which is subjectively linearly related to the log of the viscosity of the carboxymethylcellulose (Kadohisa et al. 2005a). The nonfat oils, mineral (paraffin) oil which is a pure hydrocarbon, and silicone oil (Si(CH_3_)_2_O)_n_, were included in these investigations to investigate whether neurons categorized as responding to fat also responded to nonfat oils (Verhagen et al. 2003, 2004; Kadohisa et al. 2005a, 2005b).

**Table 1:** Stimuli.

Stimulus	Abbreviation	Concentration	MWt	Approx viscosity (cP)^b^	Chemical group	CSF^c^ 25 mm/s
Glucose	G	1 M	180	1	Monosaccharide aldohexose	
Black currant juice	BJ	20%		1	Mixture	
Monosodium glutamate	M	0.1 M	187	1	Amino acid salt	
NaCl	N	0.1 M	58	1	Inorganic salt	
HCl	H	0.01 M	36	1	Inorganic acid	
Quinine HCl	Q	0.001 M	387	1	Alkaloid	
Water	V1 or 1 cP	5 mM NaCl		1		0.169
CMC^a^	C10 or 10 cP	0.2 g + 1 l V1	70 000	5	Polysaccharide	0.110
CMC^a^	C100 or 100 cP	4.0 g + 1 l V1	70 000	108	Polysaccharide	0.076
CMC^a^	C1000 or 1000 cP	11.0 g + 1 l V1	70 000	945	Polysaccharide	0.057
CMC^a^	C10000 or 10 000 cP	24.0 g + 1 l V1	70 000	8550	Polysaccharide	0.035
Mineral oil	MO	100%		26	Hydrocarbon mixture	0.031
Silicone oil	SiO or SilO	100%		10 100, and 1000 or 280	Silicon-oxygen Polymer	0.007 for Si280
Vegetable oil	VOo or VOf	100%		56	Fat	0.029
Coconut oil	CO	100%		118	fat	0.032
Safflower oil	SaO or SafO	100%		50	fat	0.035
Single cream	SC	100%		250	emulsion	0.031
Lauric acid C12:0	LaA	100 μM		1	FFA	0.11
Linoleic acid C18:2	LiA	100 μM		1	FFA	0.11

^a^CMC—carboxy-methyl-cellulose.

^b^The values given for the viscosity are those measured with the Kinexus Rheometer rather than the nominal values measured previously with a Brookfield rheometer.

^c^CSF—coefficient of sliding friction.

Sixty eight of the neurons analyzed here were in the macaque insular primary taste cortex (with the recording sites shown in Fig. 10 of Verhagen et al. (2004)), 51 in the orbitofrontal secondary taste cortex (with the recording sites shown in Fig. 11 of Verhagen et al. (2003)), and 45 in the amygdala (with the recording sites shown in Fig. 7 of Kadohisa et al. (2005b)).

### Tribology

The tribology of the stimuli was measured with an MTM2 Mini Traction Machine (PCS Instruments) using a Stribeck series with a 2 N normal force, a rolling stainless steel ball with a silicone disk rotating with a slide-roll ratio of 50%, and average of 1–1500 mm/s (Malone et al. 2003). All measurements are presented by averaging a series of six increasing and decreasing speed sweeps completed in triplicate. From these measurements, we took values at 10, 25, and 80 mm/s, as 40–250 mm/ s is thought to be representative of conditions in the mouth from previous work using a mixed lubrication regime (Malone et al. 2003). Unless otherwise stated results are presented for 25 mm/s as the correlations between the friction measures were very high (*r* ≥ 0.985) for this set of stimuli within the mixed regime. (For the cream emulsion, the Stribeck curve measures were restricted to low velocities and the results were for the first run, to minimize instabilities of the cream during measurements.)

The measurements of the CSF measured with this precise method replace what was referred to in the Discussion in a previous paper (Verhagen et al. 2003).

### Rheology

The rheology of the stimuli was measured with a Kinexus Rheometer (Malvern Instruments, UK) by subjecting a sample to a set of shear rates at values between 0.1 and 100/s while recording the viscosity at each targeted shear after reaching steady state. The values at 12/s are presented in this study, chosen as this is thought to be representative of conditions in the mouth (Shama and Sherman 1973), but the values for this set of stimuli were very highly correlated (*r* = 0.995 for the logs of the viscosities) for 50/s (Wood 1968). It has also been suggested that a shear rate lower than 1/s may be appropriate, at least for some stimuli (Li et al. 2017). However, at least for the carboxymethylcellulose used here, there is a high correlation between the log of the viscosity measured at the shear rate of 12/s and subjective thickness ratings (Kadohisa et al. 2005a), so the value of 12/s used here is reasonable. (For fatty oils, these issues will make little difference as they behave in a Newtonian way.) These rheology measurements considerably extended the original measurements with the Brookfield rheometer, and all the viscosity measures included here are those measured as just described. However, the more exact viscosity values shown in Table 1 measured as described here are close to the original nominal values shown in Table 1 that were measured with a Brookfield rheometer, and the previous conclusions about the responses of neurons related to viscosity (Rolls et al. 2003; Verhagen et al. 2003, 2004; Kadohisa et al. 2005a, 2005b) are not changed by the new more exact measurements described here. The measurements were made at 23 °C, which was the temperature of the stimuli in the original neurophysiological experiments.

### Data Analysis

The Pearson correlation between the firing rate of each neuron and 1) the CSF, and 2) the viscosity was calculated to show to what extent the firing of a neuron reflected one or other of these measures. Linear regression lines are shown in the Figures for how the firing rates were related to the CSF, or to the log of the viscosity. (The log of the viscosity was used because human psychophysical measures of the thickness of these stimuli were linearly related to the log of the viscosity (Kadohisa et al. 2005a).) Peristimulus histograms of the neuronal responses and raster plots are provided in the publications of the original neurophysiological investigations to illustrate the selectivity and dynamics of the neuronal responses (Verhagen et al. 2003, 2004; Kadohisa et al. 2005a, 2005b). The responses of the neurons described here, including those recorded in the amygdala and a comparison between brain regions, have been described in detail in these publications (Verhagen et al. 2003, 2004; Kadohisa et al. 2005a, 2005b).

### Neuronal Population Analysis: Multiple-cell Information Analysis to Analyze the Information Encoded About the CSF and About Viscosity by Different Neuronal Populations

A neuronal population analysis was performed to assess how much information from the different categories of neurons described here was available about the CSF, and about viscosity. If one category of neuron encoded, as a population, information about the CSF but not about viscosity, and another category of neuron encoded information as a population about viscosity and not about the CSF, then this would provide evidence that these two physical dimensions of the stimuli could be encoded differently in the brain. Further, it would be of interest if the neurons that responded to fat were in the category of neurons that encoded information about the CSF but not about viscosity (which was in fact the result found).

A multiple-cell Shannon information measure, the average amount of information that is obtained about which stimulus was shown from a presentation of a stimulus from the responses of all the cells in a neuronal population was implemented. If the information increases with the number of cells, then the information encoded by each cell is partly independent of that encoded by the other cells (Rolls et al. 1997). If the information saturates at one cell, then the information encoded by the set of cells is redundant with respect to each other (Rolls 2016a).

Procedures for calculating the multiple-cell information measure are given by Rolls et al. (1997). The multiple-cell information measure is the mutual information *I*(*S*,**R**), that is, the average amount of information that is obtained from a presentation of a stimulus about the set of stimuli *S* from the responses of all the cells. For multiple-cell analysis, the set of responses, **R**, consists of response vectors comprised by the responses from each cell.

Ideally, we would like to calculate

(1) $$I(S,R)=\underset{s\in S}{\sum }P\left(s\right)I(s,R)$$

However, the information cannot be measured directly from the probability table *P*(**r**,*s*) embodying the relationship between a stimulus *s* and the response rate vector **r** provided by the firing of the set of neurons to a presentation of that stimulus. This is because the dimensionality of the response vectors is too large to be adequately sampled by trials. Therefore a decoding procedure is used, in which the stimulus *s*’ that gave rise to the particular firing rate response vector on each trial is estimated. This involved for the analyses described here Bayesian maximum likelihood estimation with Poisson distributions of the firing rates of each neuron. For example, given a response vector **r** to a presentation of a stimulus, its similarity to the response vector of each neuron to each stimulus is used to estimate which stimulus was presented. The probabilities *P*(*s*’) of the current stimulus being each of the stimuli can be estimated in this way. Details are provided by Rolls et al. (1997). A probability table is then constructed of the real stimuli *s* and the decoded stimuli *s*’. From this probability table, the mutual information is calculated as

(2) $$I(S,S')=\underset{s,s'}{\sum }P(s,s')\log_{2}\frac{P(s,s')}{P\left(s\right)P(s')}$$

## Results

Correlations between the firing rates of 164 neurons and the two physical measures, the CSF and the viscosity, were calculated. All these neurons had statistically significant responses to taste, oral texture, and/or temperature (Kadohisa et al. 2005a). Sixty-eight of the neurons were in the macaque insular primary taste cortex (Verhagen et al. 2004), 51 in the orbitofrontal secondary taste cortex (Verhagen et al. 2003), and 45 in the amygdala (Kadohisa et al. 2005b). Rastergrams and peristimulus time histograms to illustrate the responses of a fat-responsive neuron recorded in the orbitofrontal cortex (Verhagen et al. 2003) are shown in the Supplementary Material as Fig. S1. The neurons that had responses to fats were categorized into the following three categories depending on the correlation of their responses with the CSF. A fourth category of neurons then described did not have preferential responses to fat but did respond to a different property of oral stimuli, their viscosity, the log of which is linearly correlated with subjective thickness (Kadohisa et al. 2005a). This fourth category was included to provide evidence on whether the relation between the oral fat sensitivity of neurons is specifically correlated with the CSF and not viscosity, whereas a separate (fourth) class is related to viscosity and food thickness, but not to fat or the CSF. It is noted that the number of neurons sensitive to the CSF (across the three categories described next) is 47/164 orally responsive neurons, which is a substantial proportion (and also higher than the relatively few neurons classified as sensing the fatty acids, LiA or LaA (17/140 tested), as noted in the Discussion and elsewhere (Kadohisa et al. 2005a)).

### Neurons with Responses Linearly Correlated with Decreases in the CSF: Linear Fat Texture Neurons

**Figure 1.**
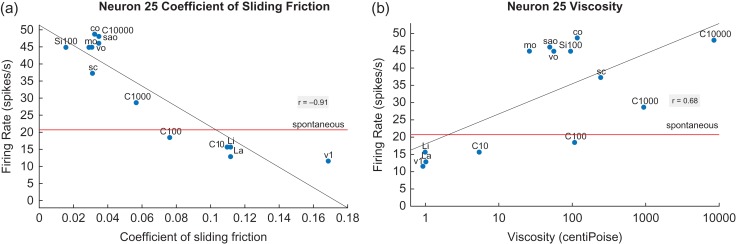
An orbitofrontal taste cortex neuron with responses linearly correlated with decreases in the CSF (a). Low CSFs indicate lubricity produced, for example, by the oils. The linear regression line shown has a correlation of *r* = −0.91 (*P* = 1.2 × 10^−5^) between the firing rate of the neuron and the CSF. The closeness of the data points to this regression line and the value of the correlation coefficient indicate how well the data fit a linear function of the CSF. (b): There is a much weaker relation to viscosity (*r* = 0.68, *P* = 0.01), with the oils producing a larger response than predicted linearly. Further, a regression line through the nonoil stimuli would have a much lower slope. C10–C10 000: carboxymethylcellulose with the nominal viscosity of 10, 100, 1000 and 10 000 cP. v1: water (1 cP). co: coconut oil; mo: mineral oil; sao: safflower oil; vo: vegetable oil; sc: single cream. Si10, Si100, Si1000: silicone oil with the viscosity indicated. LiA: linoleic acid; LaA: lauric acid. The horizontal line indicates the spontaneous firing rate. The Pearson correlation between the firing rate of each neuron and (a) the CSF, and (b) the viscosity was calculated to show to what extent the firing of a neuron reflected one or other of these measures. Linear regression lines are shown in the figures for how the firing rates were related to the CSF, or to the log of the viscosity. (The log of the viscosity was used because human psychophysical measures of the thickness of these stimuli were linearly related to the log of the viscosity (Kadohisa et al. 2005a).) Each firing rate value shown in the Figure and used in the statistical analyses presented in detail in the Supplementary Material is the mean of four or more firing rate measurements taken in random permuted sequence across the set of stimuli, with standard errors shown in the original publications (Verhagen et al. 2003, 2004; Kadohisa et al. 2005a; 2005b). Moreover, it has been established that some cortical neurons respond to water in the mouth; and that some neurons can respond to oral stimuli by decreasing their firing rates below the spontaneous level of firing (Scott et al. 1986; Rolls et al. 1990; Yaxley et al. 1990; Verhagen et al. 2003, 2004; Kadohisa et al. 2005a, 2005b).

A neuron with its firing rate responses linearly correlated with decreases in the CSF is shown in Figure 1 (left). Low CSFs indicate lubricity produced, for example, by the oils. There is a much weaker relation to viscosity (right), with the oils producing a larger response than predicted linearly. Further, a regression line through the nonoil stimuli would have a much lower slope.

Seven neurons with similar properties were recorded (4/68 in the insular primary taste cortex, 1/51 in the orbitofrontal secondary taste cortex, and 2/45 in the amygdala), with full details of their coefficients provided in the Supplementary Material in Table S1.

### Neurons with Responses Nonlinearly Correlated with Decreases in the CSF: Nonlinear Fat Texture Neurons

**Figure 2.**
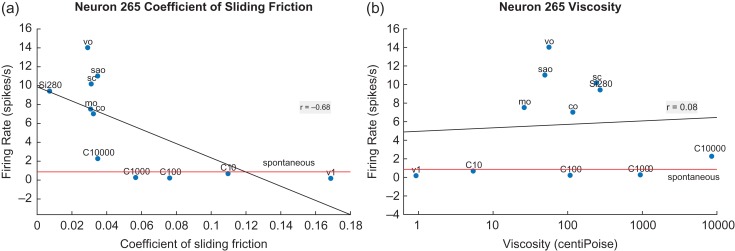
An orbitofrontal cortex (secondary taste cortex) neuron with responses nonlinearly correlated with decreases in the CSF (a). The neuron responds almost not at all until the CSF falls below 0.04. The neuron is thus very selective for fat texture, because of its nonlinear response in relation to the CSF. The linear regression line has a correlation of *r* = −0.68 (*P* = 0.02). (b): There is a much weaker relation to viscosity (*r* = 0.08, *P* = 0.82), with the oils producing a larger response than predicted linearly. Further, a regression line through the nonoil stimuli would have a lower slope. Conventions as in Fig. 1. Si280: silicone oil with a nominal viscosity of 280 cP.

A neuron with its firing rate responses nonlinearly correlated with decreases in the CSF is shown in Figure 2 (left). The neuron only responded to very low CSFs, that is, its responses were supralinearly related to decreases in the CSF. This made it a highly selective fat-encoding neuron. There is a much weaker relation to viscosity (right), with the oils producing a larger response than predicted linearly. Further, a regression line through the nonoil stimuli would have a much lower slope.

Eight neurons with similar properties were recorded (2/68 in the insular primary taste cortex, 5/51 in the orbitofrontal secondary taste cortex, and 1/45 in the amygdala), with full details of their coefficients provided in the Supplementary Table S2. Because of the nonlinearity of these neurons, a quadratic fit of the firing rates with the CSF is also provided in Table S2, together with the responses of another neuron to illustrate the better quadratic than linear fit in Supplementary Figure S1.

### Neurons with Responses Correlated with Increases in the CSF: Neurons That are Inhibited by Fat

**Figure 3.**
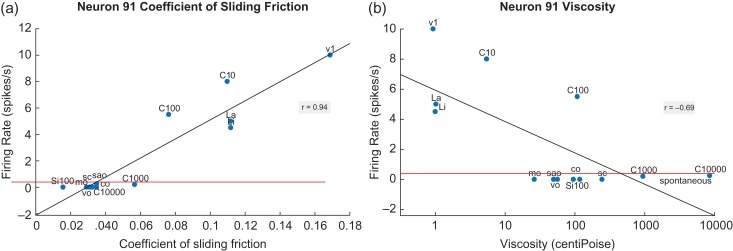
An orbitofrontal cortex (secondary taste cortex) neuron with responses correlated with increases in the CSF (a). The neuron responds almost not at all until the coefficient of linear friction is above 0.06. The linear regression line has a correlation of *r* = 0.94 (*P* = 1 × 10^−6^). There is thus no response to the fats or oils. (b): There is a weaker relation to viscosity (*r* = -0.69, *P* = 0.01), with all the oils eliciting no response. Conventions as in Figure 1.

A considerable number of neurons had low firing rates to fats and oils and higher firing rates to other stimuli. It was found that these neurons had firing rates that were correlated with the CSF. A neuron with its firing rate responses correlated with increases in the CSF is shown in Figure 3 (left). The neuron did not respond to any stimulus with a CSF less than 0.06. This made it not respond to fats and oils. This neuron had nonlinear properties and was inhibited by any stimulus with a CSF less than 0.06, including C1000 and C10 000. Some neurons had more linear responses and therefore had some response to stimuli with a CSF of 0.06, such as C1000. There is a much weaker relation to viscosity (Fig. 3 right), with the oils producing no response. This neuron responded to the LiA and LaA as predicted by their coefficients, showing that inhibition by fats and oils in this class of neuron is not produced by (at least these) fatty acids, consistent with other evidence (Rolls 2011).

Thirty-two neurons with similar properties were recorded (18/68 in the insular primary taste cortex, 6/51 in the orbitofrontal secondary taste cortex, and 8/45 in the amygdala), with full details of their coefficients provided in the Supplementary Table S3.

### Neurons with Responses to the Viscosity of Stimuli But Not to Fat

**Figure 4.**
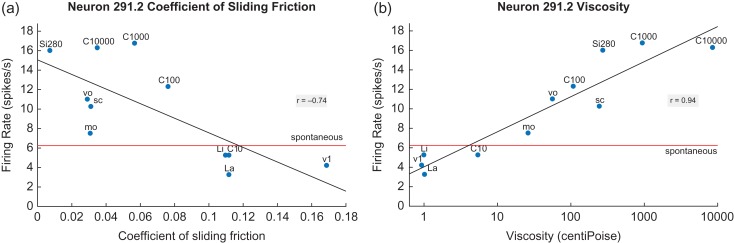
An orbitofrontal cortex (secondary taste cortex) neuron with responses correlated with increases in the viscosity of stimuli (b). The linear regression line has a correlation of *r* = 0.94 (*P* = 2 × 10^−5^). The firing rate of the neuron is less well predicted by the CSF (a) (*r* = −0.74, *P* = 0.01). Conventions as in Figure 1.

To highlight the difference of the fat-encoding and fat-inhibited neurons described above from the encoding of the thickness of stimuli in the mouth as reflected in viscosity, Figure 4 shows the responses of a viscosity-encoding neuron as functions of the CSF and the viscosity. This orbitofrontal cortex (secondary taste cortex) neuron had firing rates that were linearly correlated with increases in the log of the viscosity of the stimuli (right). The linear regression line has a correlation of *r* = 0.94 (*P* = 2 × 10^−5^). The firing rate of the neuron is not well predicted by the CSF (left) (*r* = −0.74, *P* = 0.01).

Eleven neurons with responses linearly related to the log of the viscosity were recorded (6/68 in the insular primary taste cortex, 2/51 in the orbitofrontal secondary taste cortex, and 3/45 in the amygdala), with full details of their coefficients provided in the Supplementary Table S4. Of these neurons, two had responses that were inversely linearly related to viscosity, and one neuron had responses linearly related to viscosity provided that the stimulus was not an oil. Other neurons also responded to viscosity but had tuned functions, that is, they respond optimally to a range of viscosities and not to other viscosities in the carboxymethylcellulose series (Rolls et al. 2003; Verhagen et al. 2004; Kadohisa et al. 2005a, 2005b). None of these neurons responded to the fat or fat-related oils, and this analysis was included to emphasize the point that the fat-related neurons have firing rates that are strongly correlated as a population with the CSF; and that other neurons that respond to viscosity do not respond to fat or fat-related oils. This dissociation of responsiveness helps to show that the fat-related neurons respond in relation to the CSF, but not in relation to viscosity.

### Neuronal Population Coding of Oral Fat Texture by the CSF

**Figure 5.**
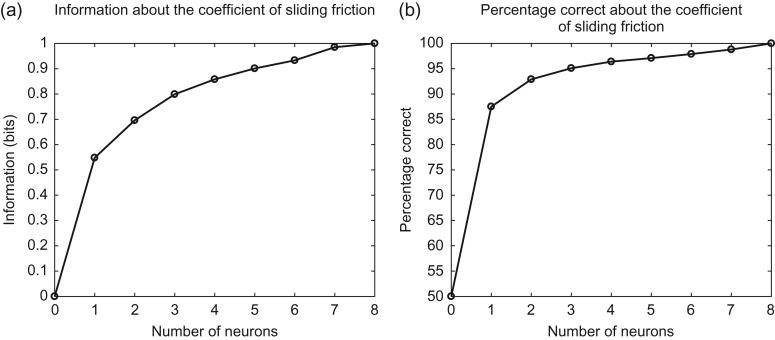
Population coding of oral fat texture by the CSF. The information available and the percentage correct about whether the set of stimuli had a CSF less than or greater than 0.35 from different numbers of nonlinear fat neurons.

A population analysis was performed in which the information available about the CSF from different numbers of neurons was performed, using the multiple-cell information analysis procedure described in the Methods and elsewhere (Rolls et al. 1997; Rolls and Treves 2011; Rolls 2016a). The information available and the percentage correct about whether the set of typically 14 stimuli shown in Figures 1–4 had a CSF less than or greater than 0.35 from different numbers of nonlinear fat neurons are shown in Figure 5. This value was chosen because all the fat and fat-related stimuli used in this investigation had CSFs < 0.35. The mean information available from these eight single cells was 0.55 bits about the CSF, and the mean percentage correct for the single cells was 85% correct. Figure 5 shows that as more cells were included in the multiple-cell information analysis, the information and the percentage correct gradually increased, until with eight cells the information was 1 bit (the maximum possible), and the percentage correct was 100%. (Chance performance is 50% correct.) A factor resulting in the gradual approach to the asymptote of 1 bit for the information is the approach to this ceiling of log_2_ of the number of categories (Rolls et al. 1997).

As a control, the information available from the set of 11 neurons categorized as responding to viscosity was 0.02 bits and 62.5% correct. This analysis thus shows that the population of nonlinear fat neurons encode information about the CSF, and that a different population of neurons with firing rates correlated with viscosity has almost no information about the CSF.

In another control, the information from the population of 11 neurons with firing rates related to viscosity was found to encode very little information about the CSF (0.02 bits 62.5% correct). On the other hand, as expected, they did code useful information about viscosity (whether the viscosity was greater than 100 cP or less than this) of 0.52 bits and 87.5% correct. This evidence is thus that there is a clear double-dissociation, with the neurons categorized as nonlinear fat neurons encoding much information in the population about the CSF and not viscosity, whereas the neurons categorized as having firing rates linearly correlated with viscosity had almost no information about the CSF, but useful information about viscosity.

Analyses of encoding by the other populations of neurons with firing rates correlated with the CSF were also performed. The neurons with firing rates linearly related to decreases in the CSF had a mean single-cell information about the CSF of the set of 14 stimuli of 0.25 bits, and for the population of 7 neurons 0.41 bits and 87.5% correct. The population of seven neurons again had no information about viscosity (0.0 bits and 54% correct).

The neurons with firing rates linearly related to increases in the CSF had a mean single-cell information about the CSF of the set of 14 stimuli of 0.16 bits, and for a population of 8 of these cells the information was 0.24 bits and 77% correct. Adding more cells up to 32 did not increase the information. The population of eight neurons again had little information about viscosity (0.08 bits and 67% correct), and this did not increase as more cells were added.

These analyses provide interesting evidence that the nonlinearity in the responses of that population of neurons to the CSF (as illustrated in Fig. 2) enabled that population to be the most highly selective population for oral fat texture. The selectivity of these neurons is emphasized by the fact that across this set of stimuli, the correlation between the CSF and the log of the viscosity was −0.74. Thus the neurons described here effectively decorrelated the representations of the CSF and of viscosity, to represent each much more selectively.

As further statistical assessment of the evidence that the neurons categorized as responding to the CSF were in different populations to the neurons categorized as responding to viscosity, two-way ANOVAS were performed, with one-factor CSF category versus Viscosity category, and the other within-subject factor the correlation of a neuron’s activity with the coefficient of sliding friction versus the correlation with viscosity. The data used were the correlation values for each neuron shown in Tables S1–S4. For the comparison between nonlinear CSF neurons and viscosity neurons, the interaction term was *F*(1,17) = 54.63, *P* < 10^−6^. For the comparison between linear CSF neurons and viscosity neurons, the interaction term was *F*(1,16) = 32.48, *P* < 10^−5^. For the comparison between neurons inhibited by increases in CSF and viscosity neurons, the interaction term was *F*(1,41) = 93.4, *P* < 10^−11^. Thus all three categories of (fat-sensitive) neurons with firing rates correlated with the coefficient of sliding friction (Tables S1–S3) were from a different population to those with firing rates correlated with viscosity (Table S4).

## Discussion

A completely new discovery of this investigation is that fat-responsive neurons in two primate taste cortical areas and the amygdala encode the coefficient of sliding friction of what is in the mouth. Some do so with linearly increasing firing rates, and others with supralinearly increasing firing rates as a function of the decrease of the coefficient of sliding friction. Another new discovery of this investigation is that there is also a population of neurons that has firing rates that are reduced according to the coefficient of sliding friction, that is, they respond less to oils and emulsions. Again, some are linear, and some are nonlinear, making them very selective in the reduction of their firing rate produced by fats, oils, and emulsions such as cream. These two classes of neuron, one responding to fat with firing rates increasing as the coefficient of sliding friction decreases (Figs 1 and 2), and the second class which is inhibited by fat and has increasing firing rates as the coefficient of sliding friction increases (Fig. 3), reveal an opponent process type of encoding that enables precise encoding of the coefficient of sliding friction, and thus whether there is the texture of fat in the mouth. These findings have we suggest important implications, for they open the way for the systematic development of foods with the pleasant mouthfeel of fat, but low energy, and health-promoting content.

The neuronal population analyses confirmed that there are different populations of neurons that encode the coefficient of sliding friction and viscosity. This is very interesting, for the coefficient of sliding friction and the viscosity of the set of stimuli used here were correlated with *r* = −0.74. Especially interesting were the neurons with responses nonlinearly related to the CSF, as illustrated in Figure 2, for these neurons as a consequence were very selective for the fats and fat-related oils, even showing little response to the carboxymethylcelluose food thickener at 1 000 and 10 000 cP, even though their CSF was a little lower than the other nonfat-related stimuli. Neuronal processes that can produce such nonlinear responses include competitive networks, as described elsewhere (Rolls 2016a). It may be biologically adaptive to also have neurons with responses linearly related to the CSF, for such neurons provide a flexible foundation for building other representations potentially useful in different and evolving environments, without the restricted potential of very highly selective neurons. The division between the low and high CSF stimuli used for this analysis was chosen because all the fat and fat-related stimuli used in this investigation had coefficients of sliding friction < 0.35. This thus helps to demonstrate that the sensitivity to the coefficient of sliding friction of several populations of the neurons described here provides a physical basis for the specificity of these neurons to fat and fat-related oils. It is noted that although these population analyses are useful, a key discovery reported here is that different individual neurons have different tuning to the coefficient of sliding friction, and it is this difference in the response of neurons that enables them to code for the specific details and indeed information about a wide range of stimuli (Rolls 2016a), and that this important information about what is represented in the brain cannot be captured by fMRI, which averages together the activity of very many thousands of neurons. Finally, the point is made that there are many other types of viscosity neurons than the set with responses linearly related to viscosity described here, and these other viscosity neurons make the encoding of viscosity very selective (Rolls et al. 2003; Verhagen et al. 2004; Kadohisa et al. 2005a, 2005b). We further note that in the original papers describing these populations of neurons, care was taken to show statistically that the neurons categorized as fat-responsive could not have arisen by chance (Rolls et al. 2003; Verhagen et al. 2003, 2004; Kadohisa et al. 2005b).

The results of the studies on insular, orbitofrontal cortex, and amygdala neurons (Rolls et al. 1999, 2003; Verhagen et al. 2003, 2004; Kadohisa et al. 2005b) show that fat-sensitive neurons respond not only to fats such as vegetable oil and other fatty oils in the mouth and to substances rich in fat such as cream and chocolate, but also to chemically different substances that have a similar slick or oily texture such as mineral oil (pure hydrocarbon), and silicone oil ((Si(CH_3_)_2_O)_n_). This evidence thus indicates that the mechanisms that sense fat and to which these neurons respond are sensing a physical rather than a chemical property of the stimuli. The results described here also provide evidence that the responses of fat-sensitive neurons are not based on a texture information channel that is tuned to viscosity. The results presented here show that their responses are based on a texture information channel that is tuned to the coefficient of sliding friction. Many nonfat substances can produce low coefficients of sliding friction, including polysaccharides (Mills et al. 2013), and such substances provide possible foods that mimic the mouthfeel of fat.

Gustatory mechanisms have been revealed in rat oral taste cells that may mediate a possible fat taste: the modulation of Ca^2+^ and K^+^ channels by long-chain free fatty acids (FFAs) such as LiA (Gilbertson et al. 1997; Gilbertson 1998; Gilbertson and Khan 2014). However, salivary lipase, which could release fatty acid from fat in rats to activate such a mechanism, is hardly present in humans (Gilbertson et al. 1997; Gilbertson 1998), so that this mechanism may not be important in humans. Further evidence that this chemical sensing mechanism may not be important in primates including humans is that the time course of the activation of the K-channel mechanism is very slow (Gilbertson et al. 1997; Gilbertson 1998) and does not match the rapidly developing subjective sensation of fat in the mouth. However, to test this possibility, in our studies in primates responses by the population of orbitofrontal cortex neurons to the FFAs, LiA and LaA were measured, and for most neurons responses were not found, that is, for most neurons the activity evoked by these stimuli was indistinguishable to that evoked by water (Verhagen et al. 2003). In particular, of 37 neurons tested with LaA and LiA, 34 had no significant responses compared with water. Of the three neurons that had statistically significant responses in this comparison, all three consisted of a smaller response than was obtained to water, and in two cases the statistical significance was marginal, i.e., *P* ≈ 0.05. The responses of the neuron shown in Figure 1 to LiA and LaA were slightly below the spontaneous firing rate, in contrast to the robust excitatory responses to safflower oil (45 spikes/s) and coconut oil (50 spikes/s), which are rich in LiA and LaA bound into triglycerides, providing evidence that the neurons did not respond to fats based on gustatory sensitivity to the fatty acids. To further assess whether the firing rates obtained to LaA and LiA could predict the responses of the neurons to coconut oil (high in lauric conjugated to glycerol) and to safflower (high in linoleic conjugated to glycerol), linear regression analysis was performed across the sample of 14 fat-sensitive neurons in the orbitofrontal cortex (Verhagen et al. 2003). There was no significant correlation between the responses to the fatty acids and these two fat stimuli. (For LaA, *r* = 0.45, *P* = 0.20; for LiA, *r* = 0.61, *P* = 0.06). Thus, the responses to fats by this population of neurons cannot be accounted for by sensitivity to LaA and LiA . By contrast, the responses to fats could be predicted by their response to the texture of silicone oil. (For silicone oil vs. coconut oil *r* = 0.99, *P* < 0.001; while for silicone oil vs. safflower oil *r* = 0.99, *P* < 0.001.) Together, these points of evidence (Verhagen et al. 2003) suggest that fat in the mouth can be sensed in primates independently of any oral gustatory mechanism for FFAs (the latter mechanism suggested by Gilbertson (1998) in rodents (Gilbertson and Khan 2014)). These data suggest that different sensing mechanisms and percepts are evoked by FFA as compared with fatty oils. Perceptual responses to FFA, if large enough not to also taste sour (Forss 1972), depend at least partly on the trigeminal-nociceptive pathway and may be associated with the percept of oral irritation. To the extent that fatty acid taste may occur in humans, it may tend to make food unpleasant, with a rancid flavor, and consistent with this, food manufacturers minimize the content of FFAs in foods (Mattes 2009). The oils, whether triglyceride-based or not, are sensed by a somatosensory-textural pathway and may be associated with the mouthfeel of fatty/slickness. It is the fat texture component that may impart pleasant sensory attributes to fat, as shown by the evidence that orbitofrontal cortex fat texture neurons in macaques respond less to fat texture after feeding to satiety with a high fat food (Rolls et al. 1999), with the pleasantness of oral fat represented in humans in the orbitofrontal and pregenual cingulate cortex (Grabenhorst et al. 2010).

Some of the fat-related neurons described here do receive convergent inputs from the chemical senses, in that some respond to taste (Rolls et al. 1999; Verhagen et al. 2003, 2004; Kadohisa et al. 2005a, 2005b), and some of these neurons respond to the odor associated with a fat, such as the odor of cream (Rolls et al. 1999). Some of the fat-related neurons also have oral temperature encoding inputs (Kadohisa et al. 2004, 2005b; Verhagen et al. 2004). The principle here is that information is encoded in the firing rates of neurons to different stimuli, that each neuron responds to different combinations of inputs, that the neurons encode information almost independently (up to at least reasonable numbers of neurons), and that this is a very efficient encoding scheme (Rolls and Treves 2011; Rolls 2016a). This type of encoding enables the information available to increase almost linearly with the number of neurons (Rolls et al. 2010; Rolls and Treves 2011; Rolls 2016a). This type of encoding also provides the basis for sensory-specific satiety, in that the responses of neurons that respond to a combination of taste, smell, oral texture, etc. can by adaptation implement sensory-specific satiety and thereby the effects of variety on food intake (Rolls 2014, 2015, 2016a, 2016c, 2017b, 2018). Feeding to satiety with fat (e.g., cream) decreases the responses of these orbitofrontal cortex neurons to zero on the food eaten to satiety (including its odor (Critchley and Rolls 1996)), but if the neuron receives a taste input from, for example, glucose taste, that is, not decreased by feeding to satiety with cream (Rolls et al. 1999). Thus, there is a representation of the macronutrient fat in the cortical taste and related areas, and the activation produced by fat is reduced by eating fat to satiety. It is thus the reward, affective, or hedonic value of fat that is represented in the orbitofrontal cortex (Rolls 2014, 2018). In the insular primary taste cortex, the identity of taste and not its reward value are represented, in that feeding to satiety does not reduce the neuronal responses in primates (Yaxley et al. 1988; Rolls 2016b). We do not have evidence on this for fat texture in the insula. In the pregenual cingulate cortex, where there is a taste and oral fat representation, the available evidence shows that feeding to satiety does reduce the neuronal responses to fat (Rolls 2008).

The dual or opponent process coding scheme revealed in this research, with some neurons increasing their firing rates to fat, and others having their firing rates reduced by fat, provides a robust way of representing information about the exact fat content of food in the mouth, as well as how fat is combined with other properties including taste, temperature, and viscosity. Further, the firing rates of at least the linear neurons in this investigation were somewhat monotonically related to the coefficient of sliding friction or to viscosity, illustrating that the magnitude of the variable is being represented by the firing rate, which is different to the place coding found in many other parts of the cerebral cortex (Rolls et al. 2010; Rolls and Treves 2011; Rolls 2016a, 2017a).

In terms of methodology, the tribology measurements were made with an MTM device using a silicone plate and steel ball, but were supported by measurements also made using a poly-dimethylsiloxane plate and glass ball which may more closely reflect sensing of food in the mouth, with the two sets of measurements linearly correlated with each other, and thus consistent with each other. The tribology measurements were made without saliva, but separate measurements of the properties of saliva showed that a representative value of the coefficient of sliding friction was 0.113, which is relatively high and close to that of the 10 cP carboxymethylcellulose, so that any saliva in the mouth would be unlikely to have a great effect in masking the low CSF values of fat, which would be likely to dominate. Similarly, the relatively low viscosity of saliva is unlikely with the saliva present in small proportions to greatly reduce the viscosity of very viscous solutions of, for example, carboxymethylcellulose. However, further investigation of the effects of saliva on the rheology and tribology of foods in the mouth will be of interest, though it must be remembered that saliva itself varies considerably.

Obesity is an increasing problem in the developed world with reports suggesting that more than 40% of adults are already obese or overweight (Alwan 2011). As a consequence, the food industry is attempting to reduce the calorific load of foods (Norton et al. 2006) that are consumed on a daily basis (e.g., for sauces, dressing, spreads, biscuits, cakes, chocolate etc). As fat is a major contributor to calories in foods, reduction has been investigated with some degree of success. However, this has relied on linking the tribology or viscosity to sensory data with no understanding of the way the mouth encodes the presence of fat. With this work, we have been able to link fat texture-sensitive neuron firing with friction. In addition, we have shown how the use of simple thickeners (such as carboxymethylcelluluse) can produce some change in the firing rates of the neurons as a result of sliding friction. It is therefore not surprising that simple approaches to fat reduction with hydrocolloids have not delivered products that have the required level of consumer acceptability to make it into main stream usage. The linkages discovered in this study suggests that a reduced sliding friction with low overall viscosity of the product is the target for fat reduction, and suggests that the use of fluid gels offers real potential as these systems have similar rheological and tribological properties to fat containing structures (Gabriele et al. 2010; Garrec and Norton 2012; Farrés et al. 2013; Mills et al. 2013).

In conclusion, the research described here shows that neurons encode the presence of fat in the mouth by the coefficient of sliding friction. Some other stimuli can produce the same physical effects. The neurons encode the coefficient of sliding friction in that their responses are related to that measure, with encoding also being measured by information theoretic methods which show that information can be read from the firing rates of these and similar single neurons and populations of neurons about what taste and related stimuli are present in the mouth (Rolls et al. 2010; Rolls and Treves 2011; Rolls 2016a). The discovery described here opens the way for the systematic development of foods with the pleasant mouthfeel of fat, together with ideal nutritional content. This is important, given that 30% of Americans and 10–20% of Europeans are classified as obese, with the prevalence rising in many developing countries (http://www.who.int), and that as body mass index increases, so does the relative risk of type 2 diabetes, hypertension, and cardiovascular disease (Berrington de Gonzalez et al. 2010). The discovery described here is a step toward better understanding of the brain mechanisms that contribute to the reward value of foods and the control of food intake (Rolls 2016c, 2018) and that in turn has implications for how to develop new foods with excellent nutritional content which are at the same time highly palatable. Finally, the results described here were found in macaques which provide a better model of primate central taste processing than rodents (Rolls 2014, 2015, 2016a, 2016b, 2016c, 2018) and so may be very relevant to food texture sensing in humans, and this is supported by consistent findings with fMRI in humans (de Araujo and Rolls 2004; Grabenhorst et al. 2010; Grabenhorst and Rolls 2014).

## Supplementary Material

Supplementary Data
