# Supplementary material for: The Neuronal Encoding of Oral Fat by the Coefficient of Sliding Friction in the Cerebral Cortex and Amygdala
Source: Cereb Cortex. 2018 Aug 31;28(11):4080–9. doi: 10.1093/cercor/bhy213 (PMC6188542; doi:10.1093/cercor/bhy213)
Supplement: Supplementary Data [file bhy213_supplementalmaterials.docx]

**The neuronal encoding of oral fat by the coefficient of sliding friction in the cerebral cortex and amygdala**

**Supplementary Material for Cerebral Cortex (2018) doi: 10.1093/cercor/bhy213**

**Edmund T. Rolls^1^, Tom Mills^2^, Abigail B. Norton^2^, Aris Lazidis^2^ and Ian T. Norton^2^**

**Introduction**

This supplementary material provides information about the linear correlations between the firing rate of each neuron, and the coefficient of sliding friction (r Coeff Sliding Friction) and the log of the viscosity (r Viscosity). If the correlation coefficient is p<0.05, a significance value is shown. The neurons are categorized in the way described in the main text. The Tables show the brain region within which each neuron was recorded as described elsewhere (Rolls et al. 2003; Verhagen et al. 2003; Verhagen et al. 2004; Kadohisa et al. 2005b; Kadohisa et al. 2005a), and the identifier for each neuron. The OFC is the orbitofrontal cortex.

The responses of a fat-sensitive neuron in the macaque orbitofrontal cortex are illustrated in Fig. S1 using rastergrams and peristimulus time histograms (Verhagen *et al.* 2003).

Fig. S1. Post-stimulus-time histograms and rastergrams of a fat responsive neuron (bk265) recorded in the macaque orbitofrontal cortex. Each row of the rastergram is a separate trial, run originally in random order. Each action potential of the neuron is represented by a small vertical spike. This neuron did not respond to the CMC (carboxymethylcellulose) viscosity series (labelled 1 – 1000 cP), but did respond strongly to vegetable oil, mineral oil, and silicone oil. spont is the spontaneous firing with no stimulus delivery. The stimuli were delivered into the mouth at time 0. For further information about the stimuli see Table 1. Modified from Verhagen, Rolls and Kadohisa (2003), where further information is provided about the responses of this and other neurons.

**Linear fat neurons**

The correlations of the firing rates with the coefficient of sliding friction are negative as shown in Table S1, because the firing rate increases as the coefficient of sliding friction becomes small (and the lubricity becomes higher). The correlations with the log of the viscosity are generally smaller, but for these relatively linear neurons with respect to the coefficient of sliding friction, some relation with viscosity is not unexpected, because the linear correlation between the values of the coefficient of sliding friction and the log of the viscosity is -0.72 for this set of stimuli. These neurons are clearly very different from the neurons categorized as having responses related to viscosity, which as shown in Table S4 have relatively high correlations with viscosity, and much lower, generally non-significant, correlations with the coefficient of sliding friction.

**Table S1. Linear fat neurons**

| Area | Neuron | r Coeff Sliding  Friction | r log Viscosity |
| --- | --- | --- | --- |
| OFC | 25 | -0.91 p=1.2x10-5 | 0.68 p=0.01 |
| Amygdala | 357 | -0.74 p=0.004 | 0.54 p=0.058 |
| Amygdala | 364 | -0.74 p=0.004 | 0.37 |
| Insula | 65.1 | -0.79 p=0.001 | 0.80 p=6x10-4 |
| Insula | 73 | -0.73 p=0.004 | 0.64 p=0.015 |
| Insula | 82 | -0.80 p=6x10-4 | 0.58 p=0.03 |
| Insula | 88 | -0.84 p=3x10-4 | 0.83 p=2.4x10-4 |

**Non-linear fat neurons**

In the case of the neurons in Table S2, a linear correlation with the coefficient of sliding friction underestimates the relation of the firing rate to the coefficient of sliding friction, as is illustrated in Fig. 2, and so the quadratic function with its parameters that best fits the firing rate is also shown in Table S2 (estimated with the Matlab function polyfit). An example of the quadratic fit of the firing rate to the coefficient of sliding friction is shown in Fig. S1 for another example of a non-linear fat-responsive neuron, which also clearly illustrates the independence of the firing rates of these neurons from viscosity.


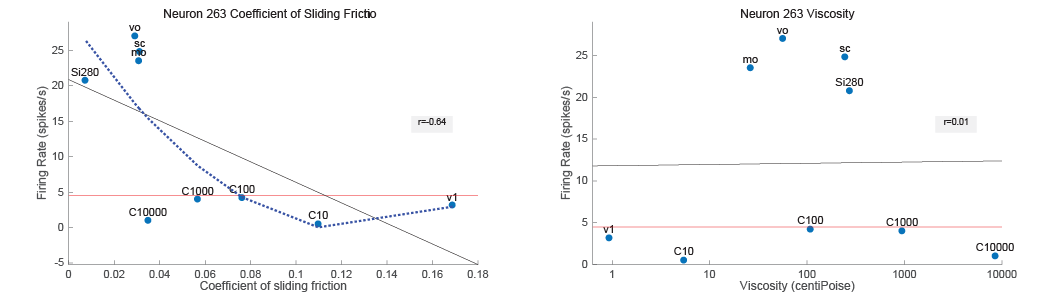


Fig. S1. An orbitofrontal cortex (secondary taste cortex) neuron with responses non-linearly correlated with decreases in the coefficient of sliding friction (CSF, left). The neuron responds almost not at all until the coefficient of sliding friction falls below 0.04. The neuron is thus very selective for fat texture, because of its non-linear response in relation to the coefficient of sliding friction. The linear regression line has a correlation of r = -0.68 (p=0.02). The quadratic regression line shown with a blue dashed line is a better fit (r=-0.86 p=0.003), but the neuron is even more non-linear than this reflects. The equation for the firing rate was 1900 s^2^ - 480 s + 30, where s is the coefficient of sliding friction. Right: There is a much weaker relation to viscosity (r = 0.01, p=0.97), with the oils producing a larger response than predicted linearly. C10-C10000: carboxymethyl cellulose with the nominal viscosity of 10, 100, 1000 and 10,000 cP. v1: water (1 cP). co: coconut oil; mo: mineral oil; sao: safflower oil; vo: vegetable oil; sc: single cream. Si280: silicone oil with a nominal viscosity of 280 cP. Conventions as in Fig. 1.

**Table S2. Non-linear fat neurons**

| Area | Neuron | r Coeff Sliding  Friction | r log Viscosity | R quadratic CSF |
| --- | --- | --- | --- | --- |
| OFC | 263 | -0.64 p=0.06 | 0.01 | 1900 s^2^ - 480 s +30 r=-0.86 p=0.003 |
| OFC | 241.2 | -0.48 | -0.05 | 644 s^2^ - 144 s + 10 r=-0.72 p=0.019 |
| OFC | 253.1 | -0.23 | -0.36 | 420 s^2^ - 103 s + 11 r=-0.84 p=0.005 |
| OFC | 265 | -0.68 p=0.02 | 0.08 | 889 s^2^ – 232 + 14 r=-0.88 p=3.1x10-4 |
| OFC | 35.2 | -0.54 p=0.06 | -0.04 | -389 s^2^ + 24 + 14 r=-0.93 p=2.8e-06 |
| Amygdala | 361 | -0.69 p=0.009 | 0.23 | 570 s^2^ - 142 s + 8 r=-0.88 p=6.1e-05 |
| Insula | 86 | -0.58 p=0.04 | 0.24 p=0.04 | 110 s^2^ - 38 s + 4 r=-0.97 pp=7x10-9 |
| Insula | 92 | -0.61 p=0.02 | 0.13 | 1036 s^2^ - 271 s + 22 r=-0.91 p=6.5e-06 |

**Neurons with responses correlated with increases in the coefficient of sliding friction: neurons that are inhibited by fat**

An example of the responses of one of these neurons is illustrated in Fig. 3. The coefficients for each neuron are provided in Table S3. The neurons generally have high linear positive correlations of their firing rate with the coefficient of sliding friction, and lower, often non-significant, correlations with the log of the viscosity, which are often but not always negative, emphasizing the independence of the coding of the coefficient of sliding friction and of viscosity described here. These neurons are clearly very different from the neurons categorized as having responses related to viscosity, which as shown in Table S4 have relatively high correlations with viscosity, and much lower, generally non-significant, correlations with the coefficient of sliding friction.

**Table S3. Neurons inhibited by fat, which have a positive correlation with the Coefficient of Sliding Friction**

| Area | Neuron | r Coeff Sliding  Friction | r log Viscosity |
| --- | --- | --- | --- |
| OFC | 73 | 0.87 p=1.3x10-4 | -0.55p=0.05 |
| OFC | 91 | 0.94 p=1.2x10-6 | -0.69 p=0.01 |
| OFC | 87 | 0.70 p=0.01 | -0.45 |
| OFC | 100 | 0.70 p=0.01 | -0.45 |
| OFC | 27 | 0.72 p=0.01 | -0.67 p<0.02 |
| OFC | 16.2 | 0.79 p=0.001 | -0.58 |
| Amygdala | 200.2 | 0.64 p=0.02 | -0.55 p=0.04 |
| Amygdala | 208 | 0.81 p=7.4x10-4 | -0.76 p=0.0015 |
| Amygdala | 213 | 0.57 p=0.04 | -0.43 |
| Amygdala | 220.2 | 0.81 p=8.4x10-4 | -0.57 p=0.03 |
| Amygdala | 223 | 0.65 p=0.02 | -0.67 p=0.01 |
| Amygdala | 239.1 | 0.86 p=1.4x10-4 | -0.65 p=0.012 |
| Amygdala | 244.2 | 0.72 p=0.006 | -0.49 |
| Amygdala | 247 | 0.80 p=0.001 | -0.63 p=0.015 |
| Insula | 157 | 0.93 p=4.8x10-6 | 0.79 p=8.4x10-4 |
| Insula | 127.1 | 0.86 p=1.7x10-4 | 0.53 p=0.05 |
| Insula | 148 | 0.90 p=2.3x10-5 | -0.74 p=0.0025 |
| Insula | 149.1 | 0.85 p=2.5x10-4 | 0.66 p=0.01 |
| Insula | 149.2 | 0.89 p=5.1x10-5 | -0.63 p<0.02 |
| Insula | 147.2 | 0.92 p=0.002 | -0.50 |
| Insula | 147.1 | 0.75 p=9.1x10-6 | -0.75 p=0.002 |
| Insula | 132.2 | 0.80 p=0.001 | -0.65 p=0.012 |
| Insula | 109 | 0.62 p=0.023 | -0.43 p=0.12 |
| Insula | 121.2 | 0.62 p=0.025 | -0.25 p=0.4 |
| Insula | 107.1 | 0.73 p=0.004 | -0.62 p=0.01 |
| Insula | 153.2 | 0.60 p=0.02 | -0.23 |
| Insula | 156 | 0.83 p=4x10-4 | -0.66 p=0.12 |
| Insula | 66 | 0.92 p=8x10-6 | -0.80 p=6x10-4 |
| Insula | 91 | 0.71 p=0.006 | -0.59 p=0.03 |
| Insula | 113.1 | 0.84 p=3x10-4 | -0.82 p=3x10-4 |
| Insula | 114.2 | 0.82 p=6x10-4 | -0.80 p=7x10-4 |
| Insula | 123.1 | 0.74 p=0.004 | -0.36 |

**Neurons with responses related to viscosity**

An example of the responses of one of these neurons is illustrated in Fig. 4. The coefficients for each neuron are provided in Table S4. The neurons generally have high linear positive correlations of their firing rate with the log of the viscosity, and low typically non-significant correlations with the coefficient of sliding friction. When contrasted with the responses of the fat-responsive neurons shown in Tables S1-S3, the responses of these viscosity-sensitive neurons emphasize the independence of the coding of the coefficient of sliding friction and of viscosity described here. Further neurons with responses related to viscosity in different ways than linearly are described elsewhere (Rolls *et al.* 2003; Verhagen *et al.* 2003; Verhagen *et al.* 2004; Kadohisa *et al.* 2005b; Kadohisa *et al.* 2005a).

**Table S4. Viscosity neurons**

| Area | Neuron | r Coeff Sliding  Friction | r log Viscosity |
| --- | --- | --- | --- |
| OFC | 291.2 | -0.39 | 0.72 p=0.004 |
| OFC | 17 | -0.25 | 0.70 p=0.008 |
| Amygdala | 181.2 | 0.52 | -0.59 p=0.03 |
| Amygdala | 202.1 | 0.52 | -0.59 p=0.03 |
| Amygdala | 262 | -0.24 | 0.71 p=0.005 |
| Insula | 132.1 | 0.40 | 0.63 p=0.015 |
| Insula | 77 | -0.64 p=0.02 | 0.69 p=5x10-4 |
| Insula | 81 | 0.45 | 0.80 p=6x10-4 |
| Insula | 112.5 | 0.42 | -0.55 p=0.04 |
| Insula | 112.2 | -0.16 | 0.62 p=0.02 |
| Insula | 113.2 | -0.39 | 0.72 p=0.004 |

References

Kadohisa M, Rolls ET, Verhagen JV. 2005a. Neuronal representations of stimuli in the mouth: the primate insular taste cortex, orbitofrontal cortex, and amygdala. Chem Senses 30:401–419.

Kadohisa M, Rolls ET, Verhagen JV. 2005b. The primate amygdala: neuronal representations of the viscosity, fat texture, temperature, grittiness and taste of foods. Neuroscience 132:33-48.

Rolls ET, Verhagen JV, Kadohisa M. 2003. Representations of the texture of food in the primate orbitofrontal cortex: neurons responding to viscosity, grittiness and capsaicin. J Neurophysiol 90:3711-3724.

Verhagen JV, Kadohisa M, Rolls ET. 2004. The primate insular/opercular taste cortex: neuronal representations of the viscosity, fat texture, grittiness, temperature and taste of foods. J Neurophysiol 92:1685-1699.

Verhagen JV, Rolls ET, Kadohisa M. 2003. Neurons in the primate orbitofrontal cortex respond to fat texture independently of viscosity. J Neurophysiol 90:1514-1525.
